# Fiber Reinforced Polymer Debonding Failure Identification Using Smart Materials in Strengthened T-Shaped Reinforced Concrete Beams

**DOI:** 10.3390/polym15020278

**Published:** 2023-01-05

**Authors:** Adamantis G. Zapris, Maria C. Naoum, Violetta K. Kytinou, George M. Sapidis, Constantin E. Chalioris

**Affiliations:** Laboratory of Reinforced Concrete and Seismic Design of Structures, Civil Engineering Department, School of Engineering, Democritus University of Thrace, 67100 Xanthi, Greece

**Keywords:** damage detection, debonding, fiber reinforced-polymer (FRP) sheet, piezoelectric lead zirconate titanate (PZT) transducers, smart sensors, shear strengthening, structural health monitoring (SHM)

## Abstract

The favorable contribution of externally bonded fiber-reinforced polymer (EB-FRP) sheets to the shear strengthening of reinforced concrete (RC) beams is widely acknowledged. Nonetheless, the premature debonding of EB-FRP materials remains a limitation for widespread on-site application. Once debonding appears, it is highly likely that brittle failure will occur in the strengthened RC structural member; therefore, it is essential to be alerted of the debonding incident immediately and to intervene. This may not be always possible, particularly if the EB-FRP strengthened RC member is located in an inaccessible area for fast inspection, such as bridge piers. The ability to identify debonding immediately via remote control would contribute to the safer application of the technique by eliminating the negative outcomes of debonding. The current investigation involves the detection of EB-FRP sheet debonding using a remotely controlled electromechanical admittance (EMA)-based structural health monitoring (SHM) system that utilizes piezoelectric lead zirconate titanate (PZT) sensors. An experimental investigation on RC T-beams strengthened for shear with EB-FRP sheets has been performed. The PZT sensors are installed at various locations on the surface of the EB-FRP sheets to evaluate the SHM system’s ability to detect debonding. Additionally, strain gauges were attached on the surface of the EB-FRP sheets near the PZT sensors to monitor the deformation of the FRP and draw useful conclusions through comparison of the results to the wave-based data provided by the PZT sensors. The experimental results indicate that although EB-FRP sheets increase the shear resistance of the RC T-beams, premature failure occurs due to sheet debonding. The applied SHM system can sufficiently identify the debonding in real-time and appears to be feasible for on-site applications.

## 1. Introduction

In the preceding decades, the pursuit of safe and resilient structures has led to an increase in criteria for reinforced concrete building design provisions. Considering that the majority of existing reinforced concrete (RC) structures are quite old and constructed in compliance with older regulations, it is challenging for them to satisfy these new, higher criteria. Furthermore, a significant portion of these structures have been subjected to multiple harsh environmental conditions or extreme loading actions, resulting in decreased bearing capacity and material deterioration.

As a result, research into strengthening and repair methodologies for RC structures has gained a great deal of interest in recent years [1]. Fiber reinforced polymers (FRPs) are commonly used for the rehabilitation of RC structures due to the material’s advantageous properties, which combine excellent environmental behavior with corrosion and chemical resistance, as well as good mechanical performance [2] which has been studied and verified through theoretical [3,4] as well experimental research. The material’s light weight and adaptability also allow for easy installation with minimal intervention to existing structural elements [5,6,7].

However, since buildings are frequently exposed to unanticipated loading actions or harsh climatic circumstances during their service life, structural degradation and deterioration may emerge in strengthened structures as well [8]. Specifically, strengthening using FRP is frequently accompanied by an unexpected and brittle failure that is primarily driven by the FRP debonding from the concrete surface.

Failure due to FRP debonding is a typical cause of failure in RC structures that have been strengthened with FRP using an external bonding technique [9,10]. Interfacial debonding at the sheet ends and interfacial debonding generated by cracks in the middle are both potential causes of FRP debonding in RC beams that have been strengthened towards flexure using FRP [11]. For RC beams that are strengthened in shear with FRP, debonding is a common failure mode, especially when U-jacketing is implemented [12,13,14]. The interfacial debonding starts when the first cracks appear and spreads throughout the structure as the cracks propagate. The bearing capacity of RC structures strengthened with FRP that have undergone debonding damages may be reduced, and in extreme cases, the structures may fail completely [15].

To prevent this failure mechanism and its disastrous effects, it would be prudent to adopt a system for field monitoring of FRP strengthened RC elements, since local debonding cannot be detected by a visual inspection alone. Such a system could be implemented in RC infrastructures where the brittle failure should be avoided at any point, such as in bridges or tunnels. Frequently in this kind of structures the strengthening using FRPs is performed in non-easily accessible areas, such as are bridge piers [16,17]. In these cases, the ability to remotely monitor the strengthened area for any signs of premature debonding is vital, as continuous on-site inspection is not possible.

Previous research has focused on detecting damage on externally bonded (EB) FRP reinforcements at an early stage in the field of strengthening RC structures with polymer composite materials [18]. Recently, the number of nondestructive approaches accessible for structural health monitoring (SHM) of EB-FRP strengthening systems has increased. Infrared thermography, ultrasonic wave velocity inspection, and X-ray inspection are some of the available SHM techniques [19,20,21,22,23,24].

Among the non-destructive, real-time SHM alternatives, the electromechanical impedance (EMI) technique appears to produce promising results [25,26]. A low-cost device, such as WiAMS, introduced by Chalioris et al. [27], combines the EMI method with piezoelectric materials and enables real-time damage detection [27,28]. Piezoelectric Lead Zirconate Titanate (PZT) transducers, a piezoceramic material with a powerful piezoelectric effect, are frequently used for SHM in the aeronautical and civil engineering fields due to their benefits of quick response, high sensitivity, wide bandwidth, dual sensing and actuating functionalities, and low cost. In the EMI technique, a PZT patch embedded or attached on the surface of a host structural member functions both as a sensor and an actuator [29,30]. Structural degradation causes a vertical or lateral shift in the baseline signatures of the structure’s initial healthy condition [27].

The use of PZT transducers for SHM with the EMI method in civil engineering fields such as artificial damages in concrete cubes [31], cracking from corrosion of steel reinforcing bars in RC members [32,33], and the heating-time effect [34] has been extensively researched. Furthermore, several studies have been conducted to determine how imposed loads affect the SHM performance of EB and embedded PZT in standard dimension specimens under compression and tension loads [35,36,37,38]. Furthermore, studies have been carried out on real-scale members such as shear critical RC beams, RC frames, and RC joints under monotonic and cyclic loading [39,40].

The recently developed technology of SHM for RC structures focuses on identifying premature debonding of FRP strengthening materials [41]. Several studies investigate the ability of PZT transducers to identify the interfacial damage between FRP laminates and concrete as flexural reinforcement in the bottom surface of the specimens [42,43,44,45]. Furthermore, experimental studies investigating the monitoring of RC beams with near surface mounted FRPs using PZTs have also been repeatedly reported in the literature [46]. Li et al. [47] studied the efficacy of the EMI technique in monitoring FRP debonding of RC beams strengthened in shear using data acquired from experiments and numerical simulations. However, investigation in this field is ongoing since there is no widely accepted system that finds extensive application. Particularly research at the level of attempting to correlate the signals received by the various systems with data obtained from the behavior of the strengthened element during loading, such as deformations of the applied FRP sheet, it is still in its infancy [48].

According to the above literature review, although research has been conducted in the direction of FRP debonding damage detection utilizing SHM systems based on a variety of methods, there is no generally accepted, reliable system that can be utilized in practice. In previous investigations, the authors proposed a wireless SHM system, which demonstrated promising results in damage detection of reinforced concrete [27,28], fiber reinforced concrete [35], and reinforced concrete strengthened with FRP ropes [41]. The successful application of this system toward the detection of damage due to FRP sheet debonding could verify to the system’s ability to identify a wide range of different types of damages and possibly lay the groundwork for its use in real structures.

Towards this direction the present paper investigates the effectiveness of this EMI based wireless SHM system to detect FRP debonding through the application of PZT sensors. For this purpose, four RC flanged beams have been constructed in order to investigate their shear behavior. Two of the beams were reference specimens with inadequate reinforcement against shear while the other two were strengthened with EB FRP sheets. PZTs transducers were attached to the strengthened beams via epoxy resin. Furthermore, strain gages were employed on the surface of the FRP sheets near the PZTs transducers to. A displacement-controlled loading sequence was selected to increase damage gradually, leading to debonding of EB FRP sheets eventually. Root mean square deviation (RMSD) was used to quantify EMI spectra changes at several displacement positions. In addition, a prompt diagnosis of debonding is attempted by strain gauges along with PZT patches.

## 2. Structural Health Monitoring and FRP Debonding Detection Technique

### 2.1. Electromechanical Impedance (EMI) Method

**Figure 1:**
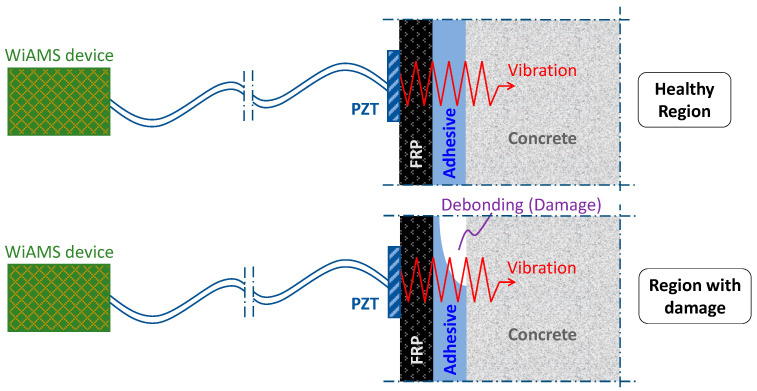
Schematic representation of FRP debonding scenario and monitoring of debonding detection.

Electromechanical Impedance technique utilizes Piezoelectric lead Zirconate Titanate transducers to exploit the electromechanical coupling effect. PZTs exhibit their characteristic property of creating a surface electric charge when subjected to mechanical stress and undergoing mechanical deformation in the presence of an applied electric field. When a PZT that is installed on a surface or embedded in a structure is actuated, damage occurrence causes a change in the structure’s mechanical impedance (or reverse admittance), resulting in variations of the electrical signal of the PZT (Figure 1). Regularly monitoring the extracted signal from the stimulating frequency of the installed PZT in a structure is an effective way to determine if structural damage has occurred. By monitoring the signatures of the piezoelectric transducers mounted on the RC element, any structural damage, such as the cracking of concrete, the yielding of steel, or debonding of external FRP, could be detected.

As part of the SHM methodology, PZT transducers are triggered within a predetermined frequency range, and their associated signals are recorded all at the same time. Initial measurements are performed on undamaged RC beams in order to record their sound condition and obtain a signature to be used as a reference (healthy state). Afterwards, measurements in the same frequency range are conducted on RC beams in various levels of possible damage. At each central frequency, PZT transducers were excited over the frequency range of 10 kHz to 250 kHz in 10 kHz increments, as described by Equation (1).

(1) $$V_{PZT}\left(t\right)=V_{E}\sin\left(\omega t\right)$$

where $V_{PZT}$(*t*) represents the excitation voltage of the PZT at any time, $V_{E}$ is the amplitude of excitation voltage, ω represents the driving voltage’s angular frequency, and *t* represents the time domain range.

**Figure 2:**
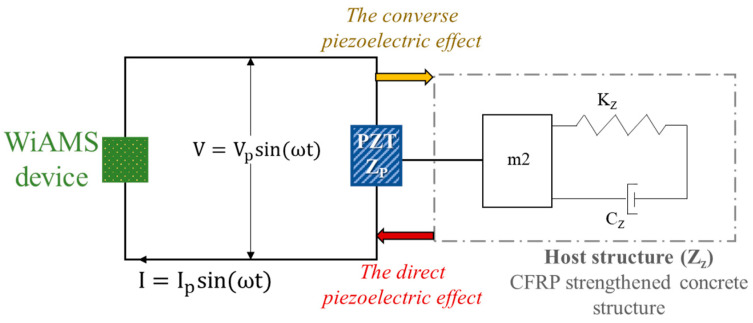
One—dimensional impedance model.

The impedance signature consisting of resistance (real part) and a reactance (imaginary part) encapsulates the interaction between PZT and the host structure. The impedance signature reflects changes in structural characteristics as a result of these interactions. Based on the dynamic equilibrium of the PZT transducer, Liang et al. [49] derived the differential equation to describe the coupling correlation as a one-dimensional impedance model (Figure 2).

(2) $$Z=\frac{\bar{V}}{\bar{I}}=R+Xj={\left[j\omega \frac{wl}{h}\left(\varepsilon_{11}^{\sigma }-d_{31}{}^{2}E_{11}^{E}+\frac{d_{31}{}^{2}E_{11}^{E}Z_{PZT}}{Z_{PZT}+Z_{S}}\frac{\tan kl}{kl}\right)\right]}^{-1}$$

In Equation (2), Z represents the overall impedance, $Z_{PZT}$ is PZT’s impedance, and $Z_{S}$ is the host structure impedance. $\bar{V}$ is the harmonic alternating voltage supplied to the circuit, $\bar{I}$ is the current passing through PZT. R is resistance, X is the reactance, and j is the imaginary unit. The patch’s width, thickness, and length are indicated by w, h, and l, respectively.$\;\varepsilon_{11}^{\sigma }$ is the complex dielectric constant, $E_{11}^{E}$ is the complex modulus under zero electric field and $d_{31}$ is the piezoelectric strain coefficient of the PZT. Additionally, the coefficient k relates to the excitation frequency ω, according to the following equation.

(3) $$k=\sqrt{\frac{\omega^{2}\rho }{E_{11}^{E}}\;}$$

Any structural damage affecting vibration transmission in the host structure, such as FRP sheets debonding, will alter the host structure’s stiffness K_s_, mass m_s_, and damping c_s_ characteristics, resulting in a change in structural parameters and a corresponding change in impedance. Consequently, based on Equation (2), as the impedance of the PZT $Z_{\mathrm{PZT}}$ remains constant, changes in the structural impedance $Z_{S}\;$will result in changes in the overall impedance Z. As a result, variations in the electromechanical impedance serve as indicators of mechanical changes in host structures.

### 2.2. Description of the Wireless Impedance/Admittance Monitoring System (WiAMS)

The EMI methodology, or reverse electro-mechanical admittance, is used as part of the developed SHM system to evaluate measurements of the output of PZT transducers, which act as both actuators and sensors on the host structure, in this study a CFRP reinforced concrete structure. The wireless impedance/admittance monitoring system (WiAMS) is used to measure the impedance magnitude of a PZT transducer in order to monitor the structural health of RC beams in real time. It includes various features, such as a remote control, high processing power, wireless data upload to a database with email notifications, and scheduled, iterative estimations of impedance magnitude within a frequency range of 5 kHz to 300 kHz with 1 Hz resolution.

The following aspects are used to estimate the magnitude of the PZT impedance from the WiAMS device. The input voltage signal can be expressed as a function of time using the following expression:

(4) $$V_{PZT}\left(t\right)=V_{P}\sin\left(2\pi ft\right)$$

where $Z_{S}$ represents the peak voltage of the signal in the frequency f. Linear systems have shifted response current signals I(t) by phase φ, with different peak currents $I_{P}$:

(5) $$I\left(t\right)=I_{P}\sin\left(\omega t+\varphi \right)$$

Since every PZT transducer exhibits nearly capacitive behavior in the presence of a pure, high-frequency sinusoidal voltage signal and tends to retain minor phase discrepancies between the voltage and current output signals, it is possible to calculate the impedance magnitude at the radial frequency by using the equation:

(6) $$V_{P}\left(\omega \right)≅\frac{\left|Z\left(\omega \right)\right|}{\left|Z\left(\omega \right)\right|+R_{f}}V_{in}\left(\omega \right)$$

This equation reveals that any variations in impedance amplitude detected in the PZT transducer are directly related to the voltage across the PZT transducer, particularly the peak voltage signal $V_{P}\left(\omega \right)$. Consequently, under steady-state conditions, if the electrical signal’s peak amplitude across the PZT transducer changes due to alterations in the structural integrity of the host structure, the voltage signal across the PZT transducer will also change. The use of this equation permits the development of simple and affordable monitoring devices in contrast to conventional and complex impedance analyzers.

## 3. Experimental Program

### 3.1. Description of Specimens

The experimental program is comprised of four beams. The beams are *L* = 2.0 m long and have a T-shape cross-section to imitate the presence of a slab. The web cross-section has a height to width ratio of *h*/*b_w_* = 300/150 mm, while the slab has a height to width ratio of *h_s_*/*b_s_* = 50/300 mm. The beams have been designed in such a way that their flexural strength exceeds their shear strength. The longitudinal reinforcement is the same for all beams and consists of 4Ø16 mm in the bottom segment of the web cross-section and 2Ø14 mm in the top. An additional 2Ø10 mm of longitudinal reinforcement has been installed in the slab area. In the shear span of two beams (S0 and S0-U), no transverse reinforcement has been installed, whereas in the shear span of the other two beams (S1 and S1-U), Ø8/400 mm stirrups have been applied in addition to the longitudinal reinforcement. Beams S0 and S1 were the reference specimens among the four beams, while S0-U and S1-U were strengthened in the shear span with FRP sheets strips. One layer of FRP sheet strips, with fiber direction perpendicular to the longitudinal axis of the beam, with a *l_s_* = 160 mm width placed at *s_s_* = 160 mm distances around the web (U-shape).

### 3.2. Strengthening Application Procedure

In beams S0-U and S1-U, beams strengthened with FRP sheets, the positions of the sheet application were pre-drawn and then the outer layer of the concrete was removed using a suitable tool. To prevent local fracture of the FRP sheets, the web section’s edges were curved. Dust and other debris were removed from the area thoroughly using compressed air. After carefully measuring and cutting the FRP sheets to the required sizes, the beam’s surface was coated with epoxy resin and the FRP sheets were meticulously impregnated with the impregnation resin, in accordance with the manufacturer’s guidelines. The sheets were then applied to the beam’s designated surface using a special roll.

### 3.3. Materials

To determine the mechanical properties of concrete, twelve cylindrical samples were cast during the concrete pouring process. Six of them were used to determine the concrete’s compressive strength, while the other six were used to determine its tensile strength. The average compressive strength of the concrete was calculated to be *f_cm_* = 44.2 ΜPa, whereas the average tensile strength was calculated to be *f_ctm_* = 3.1 MPa. Both the longitudinal and transverse reinforcements were of class B500C.

Beams S0-U and S1-U were strengthened with unidirectional carbon fiber fabric (SikaWrap^®^-600 C). According to the manufacturer, after the fiber impregnation, the nominal sheet thickness, average tensile strength, average modulus of elasticity, and elongation at rupture were 0.331 mm^2^, 3000 Ν/mm^2^, 225 kN/mm^2^, and 1.33%, respectively. The sheet was impregnated and adhered with a two-component thixotropic and epoxy resin (Sikadur^®^-300). The resin’s tensile strength, modulus of elasticity, and rupture elongation were 30 Ν/mm^2^, 4500 Ν/mm^2^, and 0.9%, respectively.

### 3.4. Experimental Setup and Instrumentation

**Figure 3:**
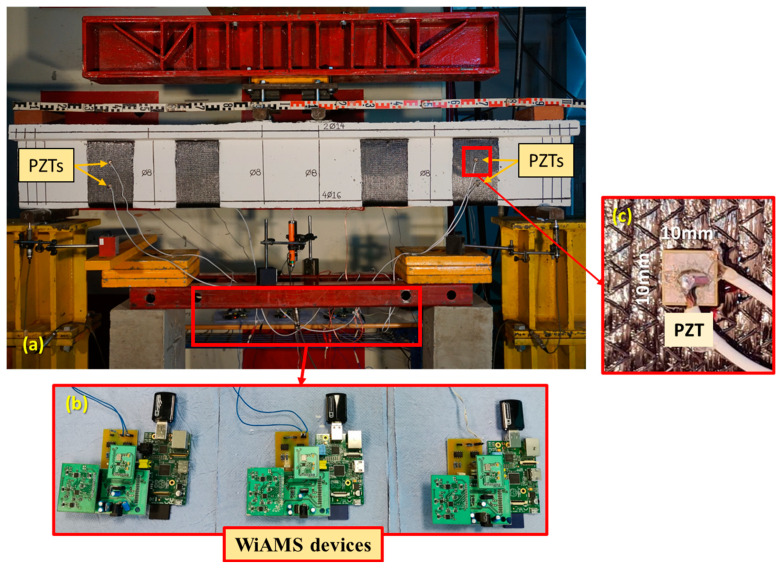
Experimental Setup and Instrumentation: (**a**) Specimen and SHM system (**b**) WiAMS devices and (**c**) PZT transducer epoxy bonded to the FRP sheet.

The specimens were supported by two metal rods positioned 100 mm from the ends of the beam. A load rate of 0.01 mm/s was applied through two metal rods, placed 100 mm on either side of the middle of the beam, in a displacement control mode. At the anticipated point of maximum moment, two linear variable differential transducers (LVDT) were positioned at the bottom of the beam (front and back sides of the beam) to measure displacements. Additionally, an LVDT was set up at the base of each support to detect any potential movement (Figure 3). Strain gauges were also installed to capture the deformations of the externally attached FRPs.

In this experimental investigation, three (3) WiAMS devices were utilized for SHM application. PZT patches were implemented to detect the debonding process of FRP sheets applied as shear strengthening of RC T-shape cross-section beams. Small-sized PI ceramics PZT transducers with dimensions of 10 mm × 10 mm (PIC 151) were used (Figure 3c). The PZT patches were carefully bonded at the surface of the FRP sheets using two-component epoxy resin. The patch poles of each PZT were connected with two cables to a WiAMS device, as shown in Figure 3b,c, to monitor the bonding degradation of FRP sheets.

**Figure 4:**
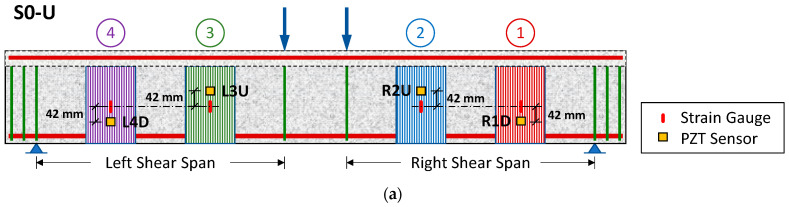

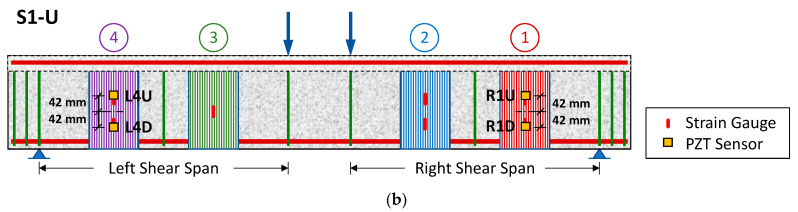
Positions and notation of the used PZT patches mounted to the FRC sheets: (**a**) of the specimen S0-U and (**b**) specimen S1-U.

In order to examine the SHM’s efficacy and accuracy in detecting the sheet debonding process, the PZT transducers have been installed in multiple positions on the surface of the EB-FRP sheets of the strengthened specimens (S0-U and S1-U). Figure 4 illustrates the notation and the position of the installed PZTs transducers epoxy bonded on the C-FRP sheets in both specimens. The PZT patches and strain gauges are denoted by three characters that vary depending on their position. The first character indicates the shear span in which the sensor is located (L or R for left or right shear span). The second character is a number that represents the numbering of the FRP sheet starting with right support (values from 1 to 4). While the third character indicates the sensor’s position on the FRP sheet relative to its centroid axis and can be U (up), M (middle), or D (down).

In both specimens, four PZT patches were carefully bonded on the FRP sheets near the strain gauges position. In particular, in specimen S0-U a PZT sensor was attached 42 mm above the center of each FRP sheet that was close to loading point (R2U and L3U). While on the sheets that were close to the supports a sensor was placed 42 mm below their middle (R1D and L4D) (Figure 4a). In specimen S1-U, all PZTs patches were carefully bonded on the FRP sheets close to the supports (Sheet 1 and 4, respectively). On each sheet a sensor was attached at 42 mm above its centroid axis and one at 42 mm below (Figure 4b).

During the loading procedure, the WiAMS devices evaluated the corresponding curves of all PZTs in specific displacements.

## 4. Results

### 4.1. Experimental Response of the Beams

In this section, the response of each beam is reported briefly by describing the progression of cracking and identifying the mode of ultimate failure. The load versus deflection at the middle of the beams diagram is presented and discussed in order to gain better understanding of the response of the beams.

During the initial stages of loading (up to a load value of 50 kN) specimen S0, the reference specimen lacking transverse reinforcement in the shear span, exhibited an elastic response. A small increase in load resulted in the appearance of the beam’s first small flexural cracks in the midspan. As the load increased further, some flexural cracks appeared in the shear spans as well. At a load of 130 kN, the first shear crack occurred in the left shear span and was immediately followed by one in the right shear span and a slight drop in beam’s strength. The shear crack in the right shear span progressively grew in length and width and extended into the slab area. The ultimate failure of the beam was eventually caused by the expansion of the slab crack (143 kN).

During the initial stages of loading, the behavior of specimen S1, a reference specimen with one stirrup in each shear opening, was similar to the one of the S0 specimen. It was characterized by a brief elastic response that was followed by the formation of a few small flexural cracks in the midspan and then in the shear spans. At a load of 124 kN, the first shear crack developed in the right shear span, while a slight loading increase after, also produced a shear crack in the left span. A further raise of the loading resulted in the appearance of a second shear crack in each shear span (156 kN and 164 kN, respectively). The width growth of the shear cracks in the left span and the concrete disorganization in the area of the slab gradually caused the beam to fail (167 kN).

The specimen S0-U, a strengthened specimen without stirrups in the shear spans, displayed initial behavior similar to the corresponding reference specimen. After the formation of flexural cracks in the midspan and shear spans, the length of which increased as the load rose, the first shear crack occurred in the two shear spans. The shear fracture occurred between the FRP sheets in the middle of the shear spans, resulting in a minor loss of strength. As the load increased, a second shear crack emerged at the intersection of the left shear span and the left end sheet. The debonding of the end FRP sheet initiated at the right shear span at a load of 176 kN. The debonding of the sheet caused a rapid increase in the width of the intersecting crack, which immediately extended into the slab region and ultimately led to the failure of the beam.

In the specimen S1-U, which had a single stirrup and strengthening in the shear spans, the flexural cracks initially appeared in the midspan and then gradually extended to the shear spans. The first shear crack appeared in the middle of the left shear span between the strengthening application. At a slightly higher load, a crack also formed at the corresponding point in the right shear opening. Shear crack length grew and a second shear crack also appeared in the right shear span as the load increased. At a load of 238 kN, the right shear span’s end sheet started to debond, which sped up the growth of the intersecting crack and led to the failure of the beam.

**Figure 5:**
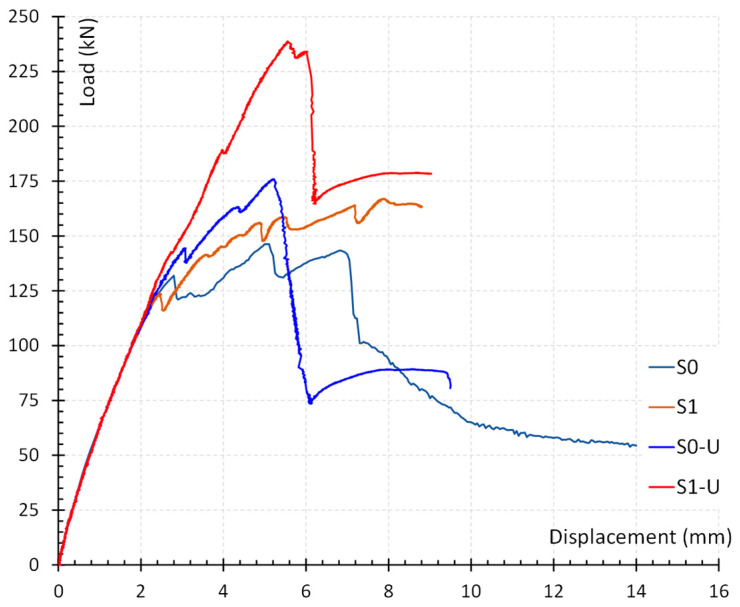
Diagram of imposed force versus deflection in the middle of each beam.

To better understand the response of the beams, a diagram of imposed force versus deflection in the middle of each beam was provided (Figure 5). Up until the formation of the first shear cracks, all four beams exhibited a similar, linear-like behavior. In the reference specimens, the initiation and propagation of shear cracks resulted in brittle failure. The presence of the stirrup in reference specimen S1 results in a less brittle failure mode than the beam without stirrup in the shear span. By installing FRP sheets as shear strengthening in the shear spans of beams S0-U and S1-U, the load-carrying capacity of the beams was increased. The strengthened beams demonstrated an almost linear behavior until the FRP sheets debonding. However, the beams’ load-bearing capacity rapidly decreases after debonding leading to the failure of the beams.

### 4.2. PZT Results

**Figure 6:**
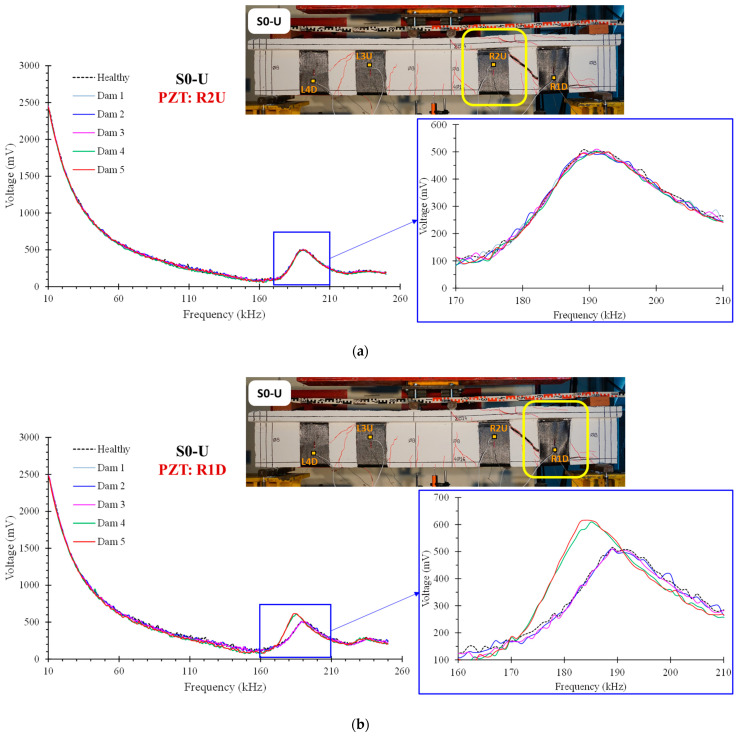
Voltage signal frequency response of PZT: (**a**) “R2U” and (**b**) “R1D”, epoxy bonded on the FRP sheets of the beam S0-U.

The typical voltage signal response curves of the PZTs “R1D” and “R2U”, that were mounted to the right shear span of the S0-U specimen, are presented and compared in Figure 6 as extracted from the WiAMS. The PZT transducer “R1D”, which was located right next to the FRP sheet’s debonding region in the right free end of the RC beam, exhibits a greater discrepancy in the final stages of damage. In addition, peak shifting (160–210 kHz) toward lower frequencies with higher amplitude is a strong indicator of debonding of FRP sheets.

**Figure 7:**
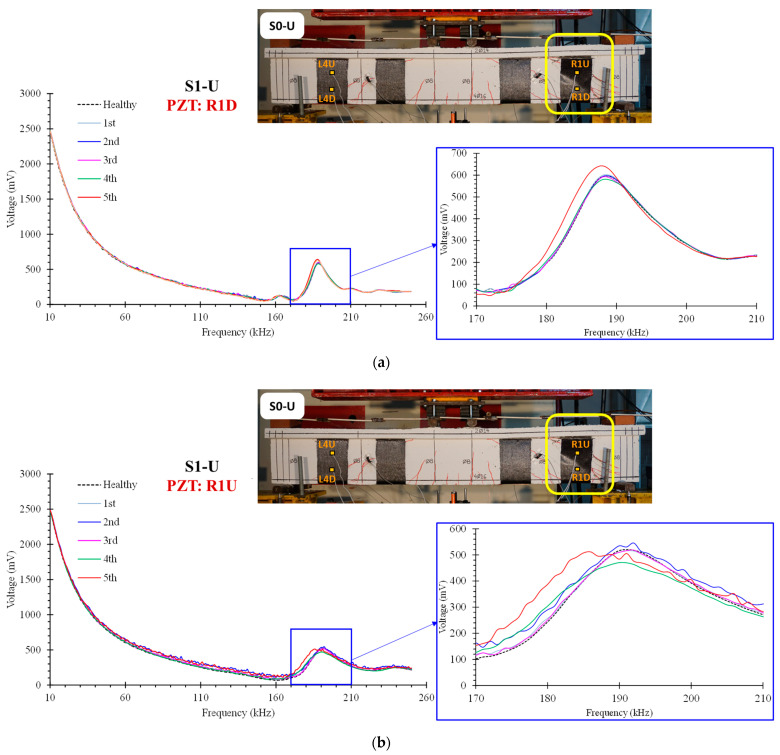
Voltage signal frequency response of PZT: (**a**) “R1D” and (**b**) “R1U”, epoxy bonded on the FRP sheets of the beam S1-U.

Figure 7 illustrates the corresponding curves of specimen S1-U from PZTs “R1U” and “R1D” mounted in the debonded FRP sheet, at the free end of the right shear span. As anticipated, both PZT measurements exhibit progressively increasing variations as a result of the FRP sheet’s debonding. The higher discrepancies observed in the measurements of PZT “R1U” are attributable to debonding that occurs primarily in the upper region of the FRP sheet.

The majority of previous studies adopts the RMSD index [3,15] to quantify discrepancies of the curves extracted from the WiAMS that function as an indicator of concrete damage or debonding of FRP sheets. The following equation can be used to calculate the RMSD index:

(7) $$\mathrm{RMSD}=\sqrt{\frac{\sum_{1}^{N}{\left({\left|V_{P}\left(\omega \right)\right|}_{D}-{\left|V_{P}\left(\omega \right)\right|}_{0}\right)}^{2}}{\sum_{1}^{N}{\left({\left|V_{P}\left(\omega \right)\right|}_{0}\right)}^{2}}}$$

where ${\left|V_{P}\left(\omega \right)\right|}_{D}$, ${\left|V_{P}\left(\omega \right)\right|}_{0}$ are the absolute values of the PZTs peaks voltage signals at the pristine condition (healthy state) and at each damage level (Dam1, Dam2, …, Dam5), respectively, and N is the number of the measurements.

**Figure 8:**
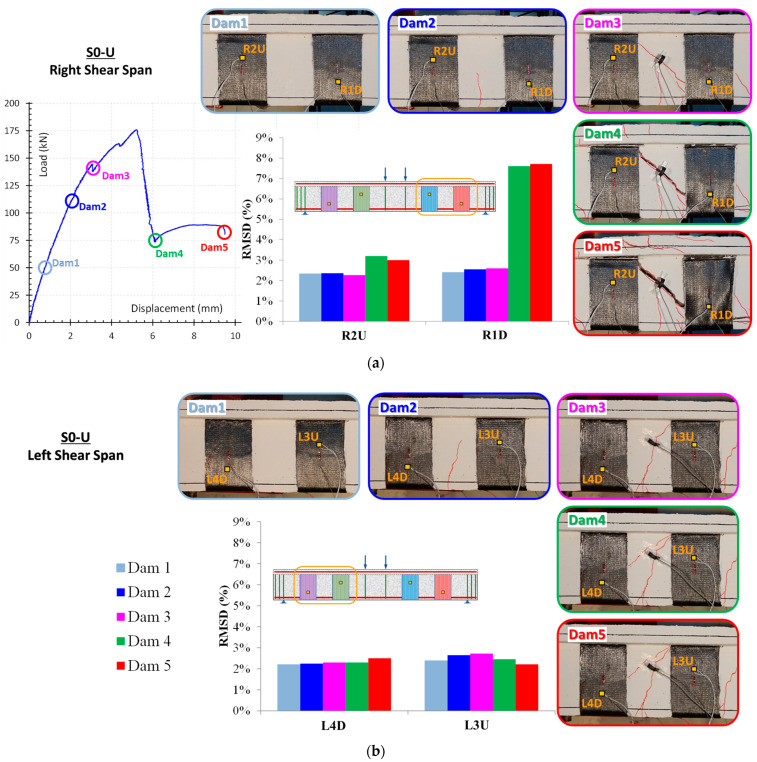
RMSD index values of PZT: (**a**) “R2U” and “R1D” and (**b**) “L3U” and “L4D”, used in S0-U specimen.

**Figure 9:**
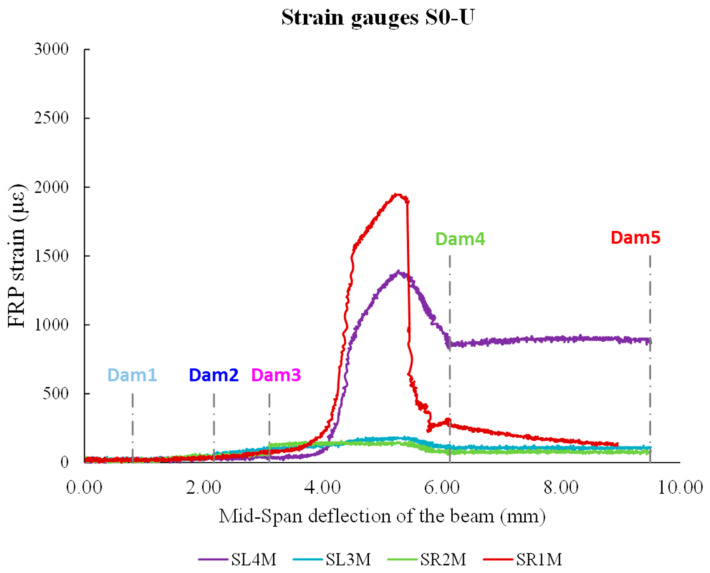
FRP sheets’ strains versus beam’s mid-span deflection in S0-U specimen.

As stated previously, the values of RMSD indices provide a scalar measure of variations in PZT signatures measured by WiAMS throughout the loading sequence. Figure 8 and Figure 9 display the RMSD indices for PZTS used in the S0-U specimen. The results indicate minor RMSD values for all PZTs at preceding damage states, until Dam 3, where slight distributed cracking occurred without evidence of bond degradation in FRP sheets.

On the contrary, the values of RMSD indices of PZTs mounted to the right shear span (“R1D” and “R2U”) show significant increases compared to PZTs mounted to the left shear span (“R4D” and “R3U”) at higher damage states (Dam4 and Dam 5). This is consistent with the specimen’s crack patterns, where the fatal crack occurred in the specimen’s right shear span. Due to the debonding of the FRP sheet on which PZT “R1D” was mounted, a significant increase in the RMSD values of PZT “R1D” has occurred. These results are consistent with the earlier assertion based on the observation of the voltage curve deviations of the PZT “R1D” (Figure 6b).

In addition, a comparison between the RMSD and strain gauge results reveals a primary finding favoring EMI-based SHM technique (Figure 9). In the early loading stages, RMSD values showed a smooth increase, contrary to the strain gauge, which showed a negligible increase. Subsequently, a sharp increase in the strain values of FRP sheets emerged between Dam3 and Dam4, leading to the debonding of FRP sheet 1 and a steep loss of bearing capacity of the S0-U specimen. During the measurements of Dam4 and Dam5, the strain values of FRP sheets decreased dramatically, whereas the RMSD values remained constant for all PZT, with the exception of the RMSD value of PZT “R1D”, which experienced an extreme increase. Consequently, the efficacy of PZTs to detect debonding of FRP sheets is established either through the observation of discrepancies in voltage curves or through the calculation of RMSD index values.

**Figure 10:**
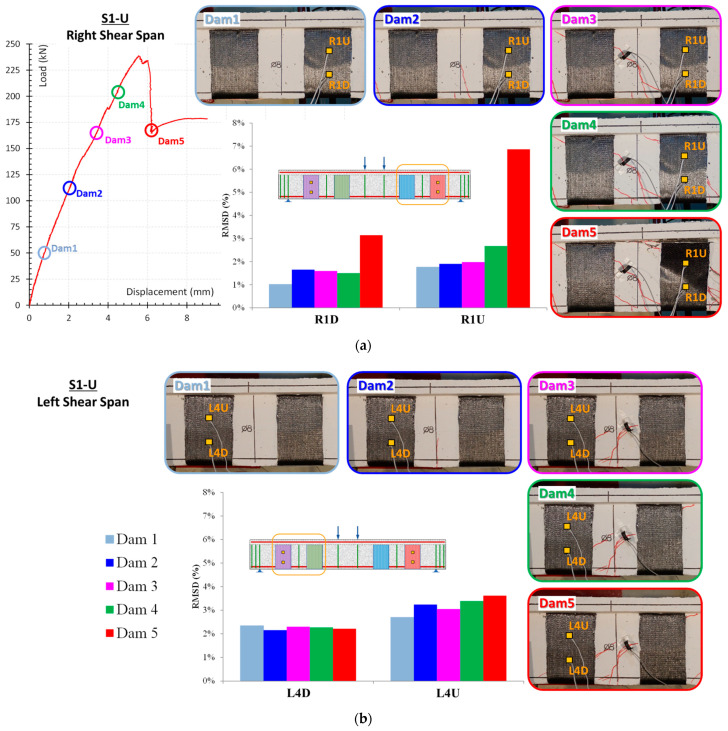
RMSD index values of PZT: (**a**) “R1U” and “R1D” and (**b**) “L4U” and “L4D”, used in S1-U specimen.

The RMSD values of specimen S1-U depicted in Figure 10 correspond to the aforementioned specimen S0-U results. Minor RMSD values are taken from all PZTs signatures measured by WiAMS in the pre-damage stages and up until Dam 4, where minimal distributed cracking has occurred in FRP sheets without indication of bond deterioration.

The loading increased until debonding occurred in the FRP sheet at the right free end, resulting in a rapid decline in the S1-U specimen’s load carrying capacity. For the PZTs “R1U” and “R1D”, the RMSD values indicate vigorous growth due to the debonding of FRP sheet 1 in Dam5. In contrast, the RMSD values of PZTs “L1U” and “L1D” indicate minor fluctuations for the examined five damage levels.

**Figure 11:**
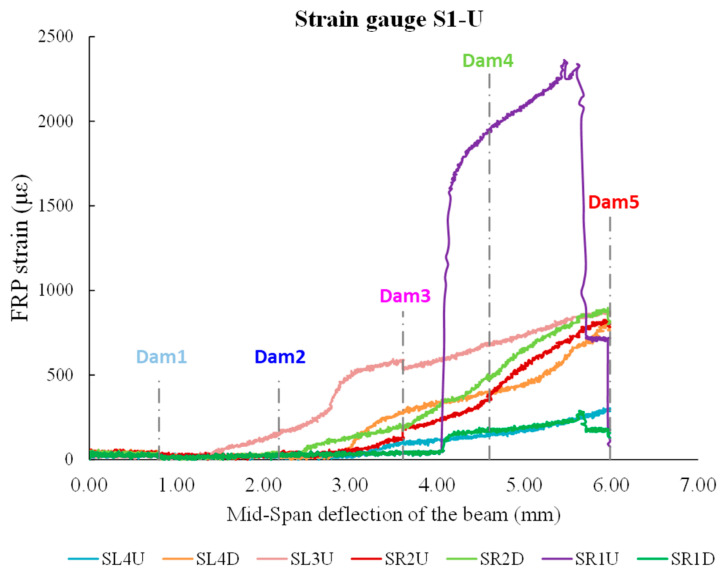
FRP sheets’ strains versus beam’s mid-span deflection in S1-U specimen.

Additionally, the RMSD results were compared to the strain gauge results (Figure 11). For the prior to damage states, the RMSD and strain gauge values increased smoothly for all PZT. The RMSD and strain gauge values of the sensors located in the upper section of the FRP sheet 1 increased more steeply between Dams 3 and 4. Subsequently, as a result of the debonding of the FRP sheet 1 in Dam5, the strain gauge values dropped significantly, whereas the RMSD values exhibited an extreme increase.

Recent studies [43,47] attributed to the detection of FRP debonding from concrete revealed that the RMSD index is the most suitable for the representation of debonding detection. This remark is supported by the findings of the present experimental study, as the RMSD index appears to reflect the debonding location as well as the moment it occurs as the index rises for the sensor that is implemented close to the debonding area.

In addition, it is evident from these recent studies [43,47] that the impedance curve remains almost unchanged and undergoes a uniform and steady variation for all sensors during the stage preceding the occurrence of damage, whereas the impedance curve undergoes significant alterations as the damage occurs. In the current investigation, authors also notice that the impedance curve follows a uniform and steady course for all sensors until debonding takes place, in the frequency range of 160–210 kHz.

Deng et al. [43] also report that the RMSD damage indices rise significantly as the load increases and clearly demonstrate the debonding of the FRP from the strengthened beam. Furthermore, the differentiation of the indices between the sensors placed in area where the damage occurs and those located far from it exceeds 35%. In the current investigation, the RMSD damage index, as shown in Figure 8 and Figure 10, increases dramatically during debonding for pzt sensors situated near the debonding area. For instance, the RMSD of the R1D sensor for DAM5 is approximately 8%, but the RMSD of the L4D sensor, located in a non-debonded region, for DAM5 is approximately 3%. As a result, the difference between the indices observed in this study is over 50%.

## 5. Conclusions

In the present study, the ability of a wireless SHM system to detect debonding of EB- FRP sheets was investigated. Specifically, experimental tests were carried out on 4 specimens of T-shaped reinforced concrete beams. Two of the specimens were reference beams, while the other two were strengthened in shear with EB-FRP sheets. In order to detect debonding in the strengthened specimens, piezoelectric sensors were externally mounted at multiple positions on the surface of the FRP sheets. Additionally, strain gauges were also attached to the surface of the sheets near the PZT sensors in order to monitor the sheets’ deformation and compare the results to those of the PZT sensors. The results of the experimental specimens’ responses were presented in the form of diagrams indicating the force versus displacement in the middle of the beam. The results of the piezoelectric sensors were presented as voltage-frequency diagrams and interpreted using the RMSD statistical index for five levels of damage. The following conclusions are drawn from the findings presented in this paper.

- The external application of FRP sheets as shear reinforcement on the web section of the beams (U-shape) increases their shear strength. However, premature debonding of FRP sheets is observed at small strain values. The FRP debonding failure has brittle nature resulting in abrupt reduction in the beams’ strength and eventually leads to its ultimate shear failure.
- The identification of FRP sheet debonding was successful, as evidenced by data collected from PZT sensors externally attached to the sheets’ surfaces. The experimental response of the specimens confirms the efficacy of the piezoelectric sensors in detecting debonding. Specifically, where a decline in strength is detected on the force-displacement diagrams of the specimens, a matching increase in the RMSD index of the sensors located close to the area of the sheet debonding is recorded.
- In addition to debonding detection, it appears that the proposed method employing piezoelectric sensors may also successfully locate its position. This is first verified visually by observing the specimen’s response during the experimental test, as shown in the related figures. The piezoelectric sensor positioned on the debonded sheet consistently displays the highest signal variation. Comparing the strain values acquired from the strain gauges with the data from the piezoelectric sensors is another indication of the ability to locate the debonding, since the strain gauges in the debonded sheet provide a sudden increase in strain that is proportionate with the significant variation in the signal of the nearest piezoelectric sensor.
- In the current investigation, reference specimens were used to determine the pattern of anticipated crack propagation regions and to ensure that the piezoelectric sensors were installed on the strengthened specimens in the most suitable locations to more accurately detect the debonding. It should be noted that the arrangement of the piezoelectric sensors has a significant impact on how accurately and effectively the presented approach can monitor the structural integrity. However, at the real-structure level, it is difficult to predict the location of the anticipated development of the damages. To achieve the best possible monitoring procedure without simultaneously necessitating the employment of a large number of sensors, additional experimental studies are required to assess the optimal arrangement network for piezoelectric sensors in real-world structures.

The ability of the suggested method to successfully identify sheet debonding is a considerable advantage, particularly for its usage in structural elements that are crucial to the structural integrity of the building and whose frequent visual inspection is impractical. Of course, the availability of a system that, in addition to detecting debonding, could also predict potential debonding at an earlier stage would be desirable. The system utilized in the present study has yielded promising results in this direction, although the research is still in its infancy, and additional study is required to develop the appropriate techniques and achieve this objective.

To further improve the proposed methodology and raise the chance of its practical application, additional study is necessary that focuses not only on the detection of damage but also on determining its extent. On this basis, an image of the complete response of the structure as well as the residual strength after debonding can be generated. A local debonding will undoubtedly affect the structure differently than a complete debonding.

The correlation between the data acquired through the signals from the sensors and the degree and type of damage is also an important research topic that requires further investigation so that the user may draw useful conclusions without considerable preprocessing of the data. The application of machine learning approaches could provide a solution in this direction.

## Data Availability

The data presented in this study are available on request from the corresponding author.
